# Squamous Cell Carcinoma of the Pancreas: A Case Report and Review of Literature

**DOI:** 10.14740/gr605w

**Published:** 2014-07-31

**Authors:** Alan Brijbassie, Edward Stelow, Vanessa M Shami

**Affiliations:** a Virginia Tech Carilion School of Medicine, Roanoke, VA 24016, USA; b University of Virginia Health System, Charlottesville, VA 22908, USA

**Keywords:** Squamous cell cancer, Pancreas, Metaplasia

## Abstract

Primary squamous cell carcinoma (SCC) of the pancreas is an extremely rare tumor with the normal pancreas being entirely devoid of squamous cells. It, however, has been noted that during inflammatory episodes, squamous metaplasia of ductal columnar cells has been observed; however, transformation to SCC is rare. We herein describe a case of pancreatic SCC and provide a review of existing literature.

## Introduction

Primary squamous cell carcinoma (SCC) of the pancreas is an extremely rare tumor with a reported incidence of 0.5-2% of all pancreatic malignancies [1-3]. The normal pancreas is entirely devoid of squamous cells; however, during inflammatory episodes squamous metaplasia of ductal columnar cells has been observed in 9-64% of cases examined at autopsy [4]. Despite this increased frequency, transformation to SCC is a rare event. Its pathophysiology thus remains as elusive as its optimal treatment with cases inexorably declining to eventual death.

## Case Report

**Figure 1:**
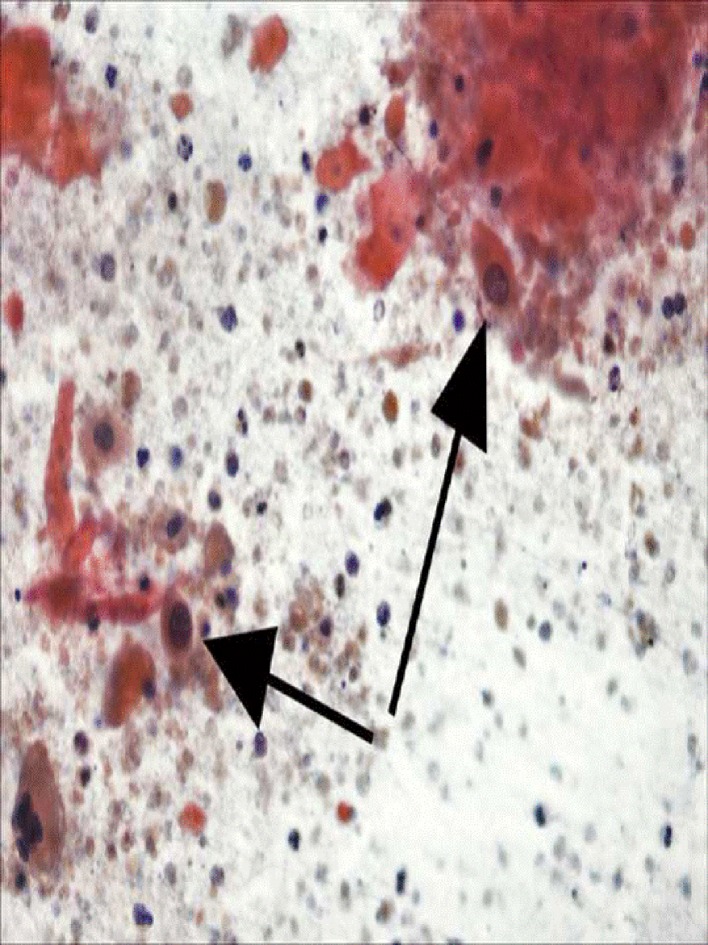
Well-differentiated SCC with smears illustrative of predominately necrotic keratinous debris with occasional malignant squamous cells.

A 60-year-old Caucasian female presented with a 1-month history of epigastric pains, anorexia, bloating and fatigue with an unintentional 10 lb weight loss over a 2-week period. Past medical history was only significant for GERD without any history of pancreatic or hepato-biliary disorders, and physical examination was significant for only mild epigastric tenderness without lymphadenopathy. EGD evaluation revealed mild chronic inflammatory changes of the esophagus; however, abdominal ultrasonography revealed a cystic lesion in the pancreatic uncinate process measuring 5 × 3.8 × 4.7 cm. Computerized tomography (CT) confirmed this lesion but was also significant for additional findings of SMV encasement by tumor as well as splenic vein thrombosis. Endoscopic ultrasonography additionally revealed proximal pancreatic ductal dilatation; fine-needle aspiration and tru-cut biopsies were subsequently sent for pathological evaluation. Laboratory values were significant for an elevated carcino-embryonic antigen (CEA) level of 1,473.5 and a cystic fluid amylase level of 7,536 U/L. Histological examination of the solid components of the lesion was confirmatory for well-differentiated SCC with smears illustrative of predominately necrotic keratinous debris with occasional malignant squamous cells (Fig. 1). Neoadjuvant chemo-radiation was commenced in the hope of possible surgical resection; however, follow-up CT revealed an interval increase in the size of the lesion, peri-tumoral tissue invasion and adjacent lymphadenopathy suggestive of metastatic spread.

## Discussion

SCC of the pancreas is a rare and somewhat controversial diagnosis with some authorities still questioning its existence as a primary entity. According to the World Health Organization classification, it represents a unique form of adenosquamous carcinoma. In a review of 6,668 cases of pancreatic exocrine carcinomas from various cancer registries between 1950 and 1985, the reported incidence of squamous and adenosquamous carcinoma was 0.005% and 0.01% respectively [1]. Of the 61 cases of pure squamous carcinoma reported between 1934 and 2004, only 26 possessed detailed clinical data [3].

The histogenesis of this poorly characterized lesion still remains elusive as the pancreas is entirely devoid of squamous cells. It is not uncommon to find squamous metaplasia of ductal columnar cells during periods of inflammation such as in pancreatitis; in fact, metaplasia has been reported in 9-64% of pancreases routinely examined at necropsy [4]. Despite this relative frequency, transformation to SCC is an extremely rare event. Four theories have been proposed [5]: 1) the presence of primitive cells capable of differentiating into either squamous or glandular types undergoing malignant change; 2) a pre-existing adenocarcinoma undergoing squamous change; 3) squamous metaplasia of the ductal epithelium during periods of inflammation with subsequent malignant transformation; and 4) an aberrant squamous cell undergoing malignant change.

Despite induced squamous metaplasia, the transformation into SCC is an unusual occurrence not only clinically but also in experimentally-induced pancreatic tumors [11]. Squamous cell contaminants can potentially contribute to diagnostic uncertainty although transgastric and transduodenal routes for EUS-FNA should not produce many of these cells. Statistically though, the presence of pure SCC in the pancreas favors a metastatic lesion until proven otherwise, and appropriate radiographic and endoscopic evaluations are needed to rule out this possibility. An autopsy series by Cubilla et al as reported by Layfield reported out of 411 neoplasms within the pancreas, 261 were noted to be metastatic with 49 originating from the lung, 12 from the cervix and 10 from the esophagus [12].

Clinical manifestations are non-specific and indistinguishable from adenocarcinoma with cholestasis, upper abdominal pains, back pains, anorexia, weight loss, nausea and vomiting being the most frequently reported [15]. No specific laboratory investigation has been helpful thus far. Minami et al reported the role of SCC antigen as a marker for tumor recurrence due to decreased values noted post resection; however, this association still requires further validation [13]. Hypercalcemia is another laboratory finding that has been reported in pancreatic SCC without evidence of bony metastatic disease thought to be related through the mediation of various humoral mechanisms including parathyroid hormone, parathyroid hormone-like peptides, prostaglandins, vitamin D-like sterols and osteoclast activating factor [16].

Radio-pathologic correlations have thus far been scant; however, Sprayregen et al reported a case displaying new vessel formation and an angiographic “tumor blush” [17]. This finding is unusual in typical adenocarcinoma and was put forth as a differentiating feature, however, by itself is non-specific and may be noted in cystadenomas, cystadenocarcinomas, islet cell tumors, angiosarcomas and hemangiomas [18].

Pre-operative histologic diagnosis at one time remained a diagnostic dilemma; however, with the advent of EUS guided tru-cut biopsies this has become more feasible.

Histological findings characteristics include keratinization with eosinophilic cytoplasm on hematoxylin and eosin staining, the formation of whorls or “pearls” with intercellular bridges and irregularly shaped nests and cords of epithelial cells [12].

Prognosis still remains dismal with most cases undergoing dissemination at the time of diagnosis. In the 26 cases reported in the English literature, only eight cases underwent curative resection. The median survival time was 7 months (range: 6 - 16 months) for those undergoing curative resection, of whom three were alive at the time of reporting at 6, 8 and 16 months respectively. The median survival time for those who did not undergo curative resection was 3 months (range: 0.25 - 9 months) [19]. Some investigators report a better response to chemoradiotherapy based on a gemcitabine regimen; however, mid- to long-term data attained thus far still suggest poor outcomes with a median survival of 2 months from the time of diagnosis which may be worse than usual adenocarcinoma of the pancreas [11, 20].

### Conclusion

Primary pancreatic SCC is such a rare event that the finding of this entity warrants an extensive workup to rule out the possibility of metastatic disease. The disease is highly aggressive, most often locally advanced or metastatic at diagnosis and poorly responsive to traditional chemotherapeutic regimens. Based on the rare incidence of this histologic subtype, diagnostic and therapeutic options will continue to remain a monumental challenge.
